# Time and age trends in smoking cessation in Europe

**DOI:** 10.1371/journal.pone.0211976

**Published:** 2019-02-07

**Authors:** Giancarlo Pesce, Alessandro Marcon, Lucia Calciano, Jennifer L. Perret, Michael J. Abramson, Roberto Bono, Jean Bousquet, Alessandro G. Fois, Christer Janson, Deborah Jarvis, Rain Jõgi, Bénédicte Leynaert, Dennis Nowak, Vivi Schlünssen, Isabel Urrutia-Landa, Giuseppe Verlato, Simona Villani, Torsten Zuberbier, Cosetta Minelli, Simone Accordini, on behalf of The Ageing Lungs in European Cohorts (ALEC) study

**Affiliations:** 1 Unit of Epidemiology and Medical Statistics, Department of Diagnostics and Public Health, University of Verona, Verona, Italy; 2 UMR1152, Department of Pathophysiology and Epidemiology of Respiratory Diseases, INSERM, Paris, France; 3 UMR1152, Faculté de Médecine, Site Bichat, Université Paris-VII (Paris Diderot), Sorbonne Paris-Cité, Paris, France; 4 Allergy and Lung Health Unit, School of Population and Global Health, University of Melbourne, Melbourne, Australia; 5 Department of Epidemiology and Preventive Medicine, Monash University, Melbourne, Australia; 6 Department of Public Health and Pediatrics, University of Turin, Turin, Italy; 7 University Hospital and INSERM, Hôpital Arnaud de Villeneuve, Montpellier, France; 8 Clinical and Interventional Pulmonology, Department of Clinical, Surgical and Experimental Sciences, University of Sassari, Sassari, Italy; 9 Department of Medical Sciences: Respiratory, Allergy and Sleep research, Uppsala University, Uppsala, Sweden; 10 National Heart and Lung Institute, Imperial College London, London, United Kingdom; 11 MRC-PHE Centre for Environment and Health, Imperial College London, London, United Kingdom; 12 Department of Pneumology, University of Tartu, Tartu, Estonia; 13 Institute and Outpatient Clinic for Occupational, Social and Environmental Medicine, University Hospital of Munich (LMU), Munich, Germany; 14 Department of Public Health, Environment, Occupation and Health, Aarhus University, Aarhus, Denmark; 15 Respiratory Department, Galdakao Hospital, OSI Barrualde-Galdakao, Biscay, Spain; 16 Unit of Biostatistics and Clinical Epidemiology, Department of Public Health, Experimental and Forensic Medicine, University of Pavia, Pavia, Italy; 17 Allergy Centre Charité, Department of Dermatology & Allergy, Charité, Universitätsmedizin Berlin, Berlin, Germany; Universita degli Studi di Ferrara, ITALY

## Abstract

**Background:**

Smoking is the main risk factor for most of the leading causes of death. Cessation is the single most important step that smokers can take to improve their health. With the aim of informing policy makers about decisions on future tobacco control strategies, we estimated time and age trends in smoking cessation in Europe between 1980 and 2010.

**Methods:**

Data on the smoking history of 50,228 lifetime smokers from 17 European countries were obtained from six large population-based studies included in the Ageing Lungs in European Cohorts (ALEC) consortium. Smoking cessation rates were assessed retrospectively, and age trends were estimated for three decades (1980–1989, 1990–1999, 2000–2010). The analyses were stratified by sex and region (North, East, South, West Europe).

**Results:**

Overall, 21,735 subjects (43.3%) quit smoking over a total time-at-risk of 803,031 years. Cessation rates increased between 1980 and 2010 in young adults (16–40 years), especially females, from all the regions, and in older adults (41–60 years) from North Europe, while they were stable in older adults from East, South and West Europe. In the 2000s, the cessation rates for men and women combined were highest in North Europe (49.9 per 1,000/year) compared to the other regions (range: 26.5–32.7 per 1,000/year). A sharp peak in rates was observed for women around the age of 30, possibly as a consequence of pregnancy-related smoking cessation. In most regions, subjects who started smoking before the age of 16 were less likely to quit than those who started later.

**Conclusions:**

Our findings suggest an increasing awareness on the detrimental effects of smoking across Europe. However, East, South and West European countries are lagging behind North Europe, suggesting the need to intensify tobacco control strategies in these regions. Additional efforts should be made to keep young adolescents away from taking up smoking, as early initiation could make quitting more challenging during later life.

## Introduction

Smoking is the main risk factor for most of the leading causes of death.[1] Every year about 1.6 million Europeans die because of smoking, making Europe the region with the greatest proportion of deaths (16%) attributable to tobacco worldwide.[2] Tobacco smoking is also a financial burden on European healthcare systems and economies.[3]

In developed countries, smoking has been associated with a loss of at least a decade of life.[4] However cessation, especially before the age of 40 years, can dramatically reduce the risk of death and significantly improve quality of life.[4] It is therefore critical to encourage all current smokers to quit in order to reduce the burden of smoking-related diseases and mortality.[2,3]

European countries have introduced various tobacco control policies (e.g. increased taxation of tobacco products, no-smoking areas, advertising bans, graphic pack warnings, and smoking cessation programs) over recent decades. Despite a general decrease in tobacco consumption and the prevalence of smoking, still more than one in four of the European population over the age of 14 years is an active smoker.[5]

Variations in smoking prevalence are determined by variations in the rates of initiation, cessation and relapse. For this reason, monitoring trends in these rates is fundamental for evaluating current tobacco control policies and for planning future strategies that can effectively tackle the tobacco epidemic.[2] Nonetheless, there is an extreme paucity of studies on smoking cessation in the general European population.

With the aim of better informing public health policy makers about decisions on future tobacco control strategies, we estimated age trends in smoking cessation in Europe for the three decades between 1980 and 2010. The present analysis compliments our previous report on smoking initiation in Europe [6].

## Methods

### Study design and population

We analysed data obtained from six multicentre studies on random samples of the general adult population carried out in Europe between 1991 and 2013, which became part of the Ageing Lungs in European Cohorts (ALEC) consortium (www.alecstudy.org).

The Global Allergy and Asthma Network of Excellence (GA^2^LEN, www.ga2len.net) is an international cross-sectional study that was carried out on people aged 15–75 years between 2007 and 2009.[7] The Italian Study on Asthma in Young Adults (ISAYA) is a postal cohort study on subjects aged 20–44 at enrolment in 1998–2000, who were followed up in 2008–2009.[8] The Genes Environmental Interaction in Respiratory Diseases (GEIRD, www.geird.org) is a cross-sectional study carried out in Italy between 2005 and 2010.[9]

The European Community Respiratory Health Survey (ECRHS, www.ecrhs.org) is an international cohort study performed on subjects aged 20–44 years at enrolment in 1991–1994.[10] In ECRHS I, random samples of subjects participating in a postal screening (stage 1) took part in a clinical interview (stage 2), where they reported information on smoking habits for the first time. The cohort of participants in stage 2 (labelled “ECRHS clinical” throughout the manuscript) was reassessed at two independent examinations during 1999–2002 in ECRHS II,[11] and during 2010–2013 in ECRHS III.[12] The Italian arm of the ECRHS (ECRHS-Italy) is the postal follow-up of the Italian participants in ECRHS stage-1, who were surveyed in 1998–2001 and 2008–2009.[13,14] The Respiratory Health in Northern Europe study (RHINE, www.rhine.nu) is a postal follow-up of the ECRHS I stage-1 participants from Scandinavian and Estonian centres, carried out in 2010–2012 (RHINE II).[15] A flow-chart describing how the three cohorts stem from the ECRHS study can be found at https://doi.org/10.1371/journal.pone.0201881.s009.

The analyses were performed on data pooled from the six studies. Subjects who had participated in more than one study or occasion were only considered once. The distribution of subjects by sample (i.e. the original centre and study where the subjects were surveyed) is shown in S1 Table. Overall, 73 samples from 53 centres in 17 countries were included, while four samples with a low participation rate (below 25%) were discarded. The samples were grouped into four regions, according to the United Nations geoscheme and tobacco epidemiology:[16] South Europe (Italy, Portugal, Spain), West Europe (Belgium, France, Germany, the Netherlands, Switzerland), North Europe (Denmark, Finland, Iceland, Norway, Sweden, United Kingdom), and East Europe (Estonia, Macedonia, Poland).

### Data on smoking

The smoking history of each subject was reconstructed retrospectively using data from postal questionnaires for all studies, in addition to data from clinical interviews for ECRHS.[Table 1] In all studies, with the exception of RHINE (where the subjects qualified themselves as “smokers” or “ex-smokers”), smoking was defined as active smoking for as long as a year (regular smoking). Life-long never smokers were excluded from the analyses. A smoker who reported not to have smoked at all in the last month was considered an ex-smoker. Age at initiation was based on the question “How old were you when you started smoking?” for all the studies. Questions on age at cessation referred to the time of stopping or the last time smoking, with a slightly different wording across studies.[Table 1] For subjects with follow-up data (ECRHS clinical, ECRHS-Italy, ISAYA), the first information available was chosen for defining age at initiation, while age at cessation was based on the last information available.

**Table 1:** Questionnaire items on smoking.

Study	Smoking status	Age at initiation	Current smoking	Age at cessation
**GA**^**2**^**LEN**	**Have you ever smoked for as long as a year?** ['YES' means at least one cigarette per day or one cigar per week for one year]	**How old were you when you started smoking?**	**Have you smoked at all in the last month?**	**How old were you when you stopped smoking?**
**RHINE**	**Are you a smoker?** (this applies even if you only smoke the odd cigarette/ cigar or pipe every week)		**Are you an ex-smoker?**	**Stopped smoking in …** (year)
**ISAYA, ECRHS-Italy, GEIRD**	**Have you ever smoked for as long as a year?** ['YES' means at least one cigarette per day or one cigar per week for one year]		**Do you now smoke, as of one month ago?**	**How old were you when you stopped smoking?**
**ECRHS** (clinical interview)	**Have you ever smoked for as long as a year?** ['YES' means at least 20 packs of cigarettes or 12 oz (360 grams) of tobacco in a lifetime, or at least one cigarette per day or one cigar per week for one year]		**Do you now smoke, as of one month ago?**	How old were you when you stopped or cut down smoking?*
**ECRHS** (postal questionnaire)	**Have you ever smoked for as long as a year?**		**Have you smoked in the last month?**	**How old were you when you last smoked?**

*only the answers from subjects who quit smoking were considered.

### Statistical analysis

Rates of smoking cessation (per 1,000/year) were calculated as the ratio between the number of incident quitters and the total time at risk, which was defined as years from initiation to cessation for quitters, or from initiation to the last survey available for active smokers. To avoid data sparseness, time at risk was left-censored at age 16 years and at 1980, and right-censored at age 60 years and at 2010.

All the analyses were stratified by sex, region, and period (1980–1989, 1990–1999, 2000–2010). Crude rates were calculated for two age groups: 16–40 and 41–60 years. In the main analyses, age-specific trends of smoking cessation rates (with 95% confidence intervals) were estimated using two-level mixed-effects models (with subjects nested into samples), with a negative binomial outcome distribution, a logarithmic link function and an offset for log(person-years). Period was included as a linear term to adjust for possible time trends of cessation rates within each decade. Age was modelled using natural smoothed polynomial lines (splines), and the number of knots (four and five for males and females, respectively) was chosen based on model fit using the Bayesian information criterion.

As the risk of relapse is high in the first year after quitting, while it drastically drops afterwards,[17] we performed a sensitivity analysis defining smoking cessation as having quitted smoking for at least two years (with person-years re-calculated accordingly), in order to exclude subjects who might have quit smoking just for a brief time before the survey.

Finally, to evaluate whether subjects who started smoking in early adolescence had a different attitude toward smoking cessation compared to those who started later, the main models were fitted including a binary indicator for age at initiation (10–15 vs. ≥16 years) as a predictor variable. In this analysis, since we found no evidence of interaction between age at initiation and period, the three decades were combined to increase the statistical power.

The statistical analyses were performed using STATA 14.2 (StataCorp, College Station, TX).

### Ethics approval and consent to participate

Ethical approval was not requested for this secondary analysis of pooled data from previous studies. In each of the original studies, ethical approval was obtained for each centre from the appropriate ethics committee. All procedures have conformed to the principles embodied in the Declaration of Helsinki. Written informed consent was obtained from participants in the clinical examinations (ECRHS clinical). In ECRHS-Italy, RHINE, GA^2^LEN, ISAYA and GEIRD, only data from postal questionnaires were used, which were voluntarily sent back giving consent to use the anonymized data.

## Results

A total of 50,228 ever smokers with complete information on sex, year of birth, age at smoking initiation and, if applicable, age at cessation were included in the analysis.[S1 Fig and S1 Table] The distribution of the participants by study and year is shown in S2 Fig. GA^2^LEN was the study that contributed the highest number of subjects (n = 22,100). Overall, 14.0% (n = 7,058) of the subjects had follow-up data.

Females represented about half of the sample, and the median age at the last interview ranged from 38 to 50 years for subjects from South and North Europe, respectively. Overall, 43.3% (n = 21,735) of the subjects quit smoking over a total time-at-risk of 803,031 person-years. The proportion of quitters was highest in North Europe (56.0%) compared to other European regions (range: 34.1% to 37.9%).[Table 2]

**Table 2:** Demographic and smoking history data of the subjects included in the analyses, by region.

	North Europe	East Europe	South Europe	West Europe	Overall
**Countries, n**	6	3	3	5	17
**Centres, n**	15	5	19	14	53
**Subjects included, n**	18,255	5,013	19,179	7,781	50,228
**Females, n (%)**	9,927 (54.4)	2,399 (47.9)	8,716 (45.5)	3,882 (49.9)	24,924 (49.6)
**Birth year** *	1958 (1940–1983)	1961 (1940–1986)	1964 (1948–1980)	1961 (1943–1986)	1962 (1942–1984)
**Year of last interview** *	2008 (2002–2011)	2008 (2007–2012)	2001 (1998–2010)	2008 (1991–2012)	2008 (1998–2011)
**Age at last interview (years)** *	50 (24–68)	47 (22–67)	38 (23–60)	43 (22–66)	43 (23–66)
**Study, n (%)**					
ECRHS clinical	2,389 (13.1)	214 (4.3)	1,764 (9.2)	3,263 (41.9)	7,630 (15.2)
ECRHS-Italy	-	-	1,808 (9.4)	-	1,808 (3.6)
RHINE	3,665 (20.1)	364 (7.3)	-	-	4,029 (8.0)
ISAYA	-	-	8,788 (45.8)	-	8,788 (17.5)
GEIRD	-	-	5,873 (30.6)	-	5,873 (11.7)
GA^2^LEN	12,201 (66.8)	4,435 (88.5)	946 (4.9)	4,518 (58.1)	22,100 (44.0)
**Quitters, n (%)**	10,224 (56.0)	1,707 (34.1)	6,854 (35.7)	2,950 (37.9)	21,735 (43.3)
**Total person-years**	309,077	89,194	287,637	117,123	803,031

* median (5^th^-95^th^ percentile)

### Trends in smoking cessation

In the 2000s, the crude cessation rates for males and females combined were highest in North Europe (49.9 per 1,000/year) compared to the other regions (range: 26.5–32.7 per 1,000/year). For males and females aged less than 40 years, cessation rates were considerably higher in the 2000s compared to the preceding two decades in all the four regions. This was especially evident in North Europe, where the cessation rates doubled, from 24 per 1,000/year in the 1980s to 53 per 1,000/year in the 2000s. On the other hand, cessation rates after the age of 40 were similar across successive periods, with the exception of North Europe where the rates doubled, from 23 per 1,000/year in the 1980s to 47 per 1,000/year in the 2000s.

Consistent results were found when looking separately at males and females.[Table 3]

**Table 3:** Crude rates of smoking cessation (per 1,000/year), number of quitters and person-years at risk in males and females by region, age group and period.

Region	Age group		Males			Females		
			1980–1989	1990–1999	2000–2010	1980–1989	1990–1999	2000–2010
**North Europe**	**16–40 years**	**rates**†	**24.7**	**31.2**	**49.8**	**23.0**	**33.4**	**56.1**
		quitters	*1*,*150*	*950*	*714*	*1*,*252*	*1*,*321*	*1*,*111*
		*py*	*46*,*594*	*30*,*445*	*14*,*336*	*54*,*329*	*39*,*553*	*19*,*808*
	**41–60 years**	**rates**†	**25.0**	**31.1**	**48.6**	**20.3**	**28.7**	**45.6**
		quitters	*227*	*667*	*979*	*171*	*623*	*1*,*059*
		*py*	*9*,*066*	*21*,*422*	*20*,*131*	*8*,*444*	*21*,*733*	*23*,*216*
**East Europe**	**16–40 years**	**rates**†	**11.8**	**16.8**	**23.5**	**9.4**	**15.1**	**30.2**
		quitters	*151*	*190*	*172*	*105*	*153*	*200*
		*py*	*12*,*845*	*11*,*312*	*7*,*320*	*11*,*210*	*10*,*146*	*6*,*619*
	**41–60 years**	**rates**†	**22.4**	**28.9**	**28.5**	**18.0**	**19.5**	**24.3**
		*cases*	*60*	*175*	*190*	*31*	*108*	*172*
		*py*	*2*,*682*	*6*,*051*	*6*,*665*	*1*,*722*	*5*,*541*	*7*,*081*
**South Europe**	**16–40 years**	**rates**†	**17.8**	**22.4**	**33.3**	**20.4**	**24.6**	**36.9**
		quitters	*961*	*1*,*376*	*649*	*930*	*1*,*256*	*610*
		*py*	*54*,*084*	*61*,*528*	*19*,*513*	*45*,*515*	*51*,*002*	*16*,*547*
	**41–60 years**	**rates**†	**30.3**	**30.1**	**33.8**	**15.1**	**21.2**	**22.2**
		quitters	*94*	*299*	*317*	*20*	*149*	*193*
		*py*	*3*,*098*	*9*,*946*	*9*,*373*	*1*,*323*	*7*,*015*	*8*,*693*
**West Europe**	**16–40 years**	**rates**†	**20.8**	**24.1**	**33.3**	**22.3**	**22.8**	**32.7**
		quitters	*459*	*304*	*253*	*461*	*295*	*300*
		*py*	*22*,*118*	*12*,*608*	*7*,*609*	*20*,*678*	*12*,*951*	*9*,*162*
	**41–60 years**	**rates**†	**32.6**	**28.0**	**32.4**	**22.9**	**24.9**	**23.1**
		*cases*	*93*	*193*	*240*	*43*	*140*	*169*
		*py*	*2*,*856*	*6*,*891*	*7*,*416*	*1*,*878*	*5*,*633*	*7*,*323*
**Overall**	**16–40 years**	**rates**†	20.1	24.3	36.7	20.9	26.6	42.6
		quitters	2,721	2,820	1,788	2,748	3,025	2,221
		*py*	135,641	115,893	48,778	131,732	113,652	52,136
	**41–60 years**	**rates**†	26.8	30.1	39.6	19.8	25.5	34.4
		*cases*	474	1,334	1,726	265	1,020	1,593
		*py*	17,702	44,310	43,585	13,367	39,922	46,313

^†^: quitters per 1,000/year. py: person-years.

**Fig 1:**
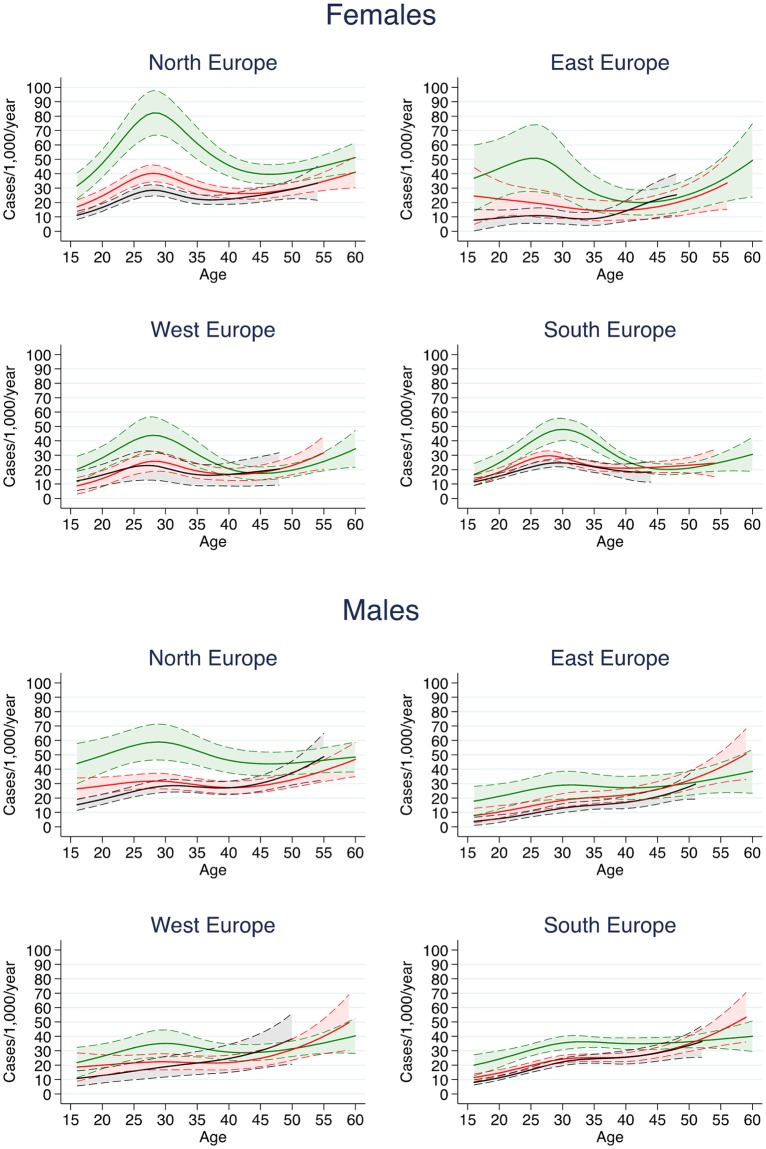
Estimated trends in smoking cessation rates (per 1,000/year) with 95% confidence intervals in females and males, by region. Trends were stratified by period: black lines refer to 1980–1989; red lines refer to 1990–1999, and green lines refer to 2000–2010.

Fig 1 shows the smoothed age-trends in smoking cessation by sex, region, and period.[Fig 1] We observed a peak in smoking cessation around the age of 30, which was particularly evident in the 2000s. This peak was quite blunted in men, while it was very sharp in women and corresponded to an age of 31 years in South Europe, 28 years in West and North Europe, and 26 years in East Europe. From the age of 40 onwards, the smoking cessation rates increased gradually in males and females from all regions.

### Smoking cessation according to age at initiation

**Fig 2:**
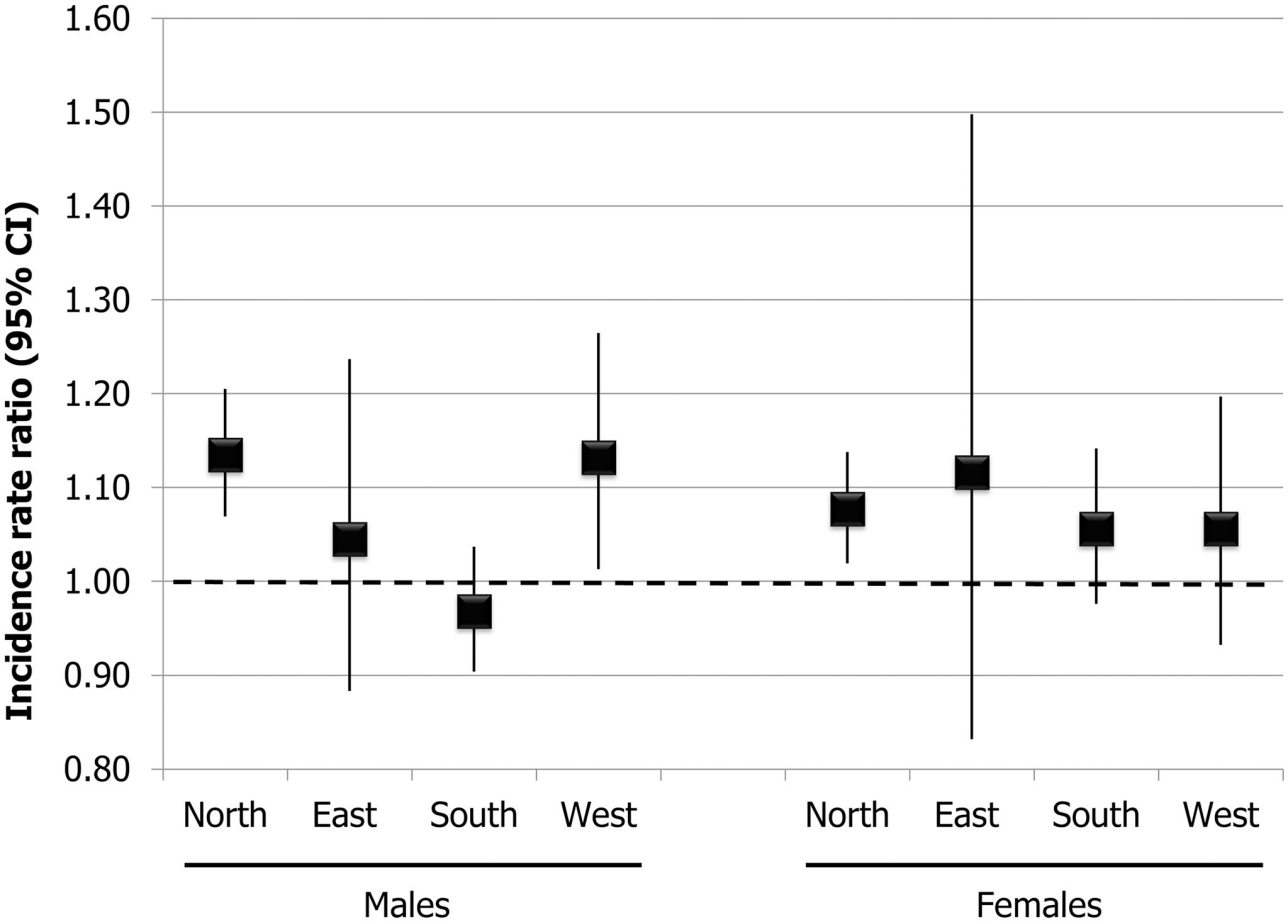
Age and period-adjusted incidence rate ratios of smoking cessation, with 95% confidence intervals, for subjects who started smoking at 16 years or later compared to those who started earlier, by sex and European region.

Except for males from South Europe, where the incidence rate ratio (IRR) for later age at initiation was below 1 and not statistically significant (IRR 0.97, 95%CI: 0.90–1.04), the likelihood of quitting was higher for both men and women who started smoking at 16 years or later compared to those who started earlier, with IRRs ranging from 1.05 (95%CI: 0.88–1.24) in East European males to 1.14 (95%CI: 1.07–1.21) in North European males.[Fig 2]

### Probability of smoking relapse after quitting

Among ex-smokers, the median duration of abstinence from smoking reported at the time of the interview was 10 years (25^th^-75^th^ percentiles: 4–18), and over 90% had quit for at least 2 years.

The risk of relapse was estimated using subjects with follow-up data.[S2 Table] Overall, 12.2% of those who were ex-smokers at the baseline examination reported to be active smokers at the last follow-up (average follow-up time: 16.5 years). The proportion of subjects relapsing was significantly greater in those who had quit smoking for less than 2 years at baseline (nearly 30%) compared to those who had quit smoking for at least 2 years (less than 10%) (p<0.001).

## Discussion

We retrospectively investigated trends in smoking cessation across three decades from 1980 to 2010, using representative samples of the general population from 17 European countries.

The main results of our analyses are the following:

- rates of smoking cessation were higher in North Europe than in West, South, and East Europe;
- smoking cessation has been steadily increasing between the 1980s and the 2000s among smokers aged under 40 years across Europe; among older people, this was observed only for North Europe;
- smoking cessation rates peak around the age of 30 in women;
- quitting was less likely for subjects who took up smoking before the age of 16 compared to people starting later.

### Time trends in smoking cessation across European regions

We showed that the rate of smoking cessation was 49.9 per 1,000/year in North Europe (United Kingdom and Scandinavian countries) in 2000–2010, which is about 60% higher compared to the other regions. Cessation rates for North Europe confirm the rates recently reported from RHINE, which is one of the cohorts included in this study,[18] while we are not aware of studies reporting cessation rates for the other regions.

We found that the rates of cessation have been increasing in North Europe since the end of the last century, while in the rest of Europe they have started to grow only after the year 2000. Moreover, in North Europe, the increasing trends in smoking cessation involved both young (16–40 years) and older (41–60 years) adults, while in the rest of Europe they were restricted to young adults. These results compliment a previous report on smoking initiation using data from the same studies, where we observed lower rates of uptake of regular smoking in North Europe compared to the other regions.[6] Overall, the geographical pattern in smoking cessation described in our study is consistent with the pattern in smoking prevalence across European countries reported by others [4, 19,20]. These data testify to the better implementation of effective tobacco control policies in countries of North Europe.[21] Of note, the higher attained education and wealth per-capita in North Europe might also have contributed to boost tobacco control strategies, as preventative measures are more effective among higher socioeconomic groups.[22]

### Age trends in smoking cessation in males and females

We found that the rates of smoking cessation were broadly similar in men and women within the same European region in the 2000s, but there was a remarkable gender difference in age trends. In fact, while the cessation rates were similar in young and older men, women aged less than 40 years had significantly higher rates than older women. These findings are in agreement with another study on population data from USA, Canada, and UK, which showed that women were more likely to have given up smoking than men before their fifties, and *vice-versa* after the age of 50.[23]

We also found a sharp peak of cessation around the age of 30 in women from all regions, which was especially evident in the 2000s. The peak slightly shifted at different ages according to the region, matching well with mothers’ mean age at first child that ranges from 26–27 years in East Europe to 30–31 years in South Europe.[24] This suggests that pregnancy is the primary reason for quitting in women. Previous reports showed that about three out of 4 women stop smoking during pregnancy,[25] and cessation is more frequent when expecting the first child.[26]

The more evident peaks of cessation in the 2000s most likely reflect an increasing awareness of the negative health impact associated with smoking during pregnancy for both the mother and the foetus,[27] and growing perception of the need to protect children from second-hand smoke exposure. Social stigma towards expecting mothers who smoke can also encourage cessation.[28] The blunted peak observed around the age of 30 among males may also be determined by parenthood, as fathers try to abstain from smoking to be supportive to their partners’ attempts to quit during pregnancy,[29,30] and to avoid their children being exposed to second-hand smoke.[31]

After the age of 40, rates of cessation gradually increased in both men and women, maybe indicating a growing concern about smoking-related illnesses with ageing.[32]

### Age at initiation affects the likelihood to quit

Although overall rates of smoking initiation have decreased considerably in Europe during recent decades, initiation in young adolescents (11–15 years) has alarmingly increased.[6] Early initiation is associated with increased risk of smoking-related mortality and morbidity.[33] Males starting smoking before age 15 may have children with an increased risk of respiratory diseases, possibly through epigenetic changes in sperm precursor cells.[34] We found that early age at initiation is associated with lower rates of cessation, in agreement with other studies from North America.[35,36] Early initiation has been associated with greater risk of relapse after cessation.[37] Younger adolescents develop nicotine addiction after a shorter duration of smoking and a lower number of cigarettes compared to those who take up smoking later.[38] All these findings suggest that tobacco control strategies targeting the youngest should be implemented with the aim of discouraging early smoking, or at least delaying initiation.

### Strengths and limitations

The main strengths of our study are the inclusion of large samples of subjects from 17 European countries and a long observation period, allowing the comparison of cessation rates across different regions and their trends during three decades.

A drawback is that this is an observational retrospective study that relies on self-reported information on smoking history, and smoking status was not verified with biochemical methods. However, the agreement between self-reported smoking and cotinine levels is generally good in population studies.[39]

Another limitation is the potential telescoping bias, as individuals might be inaccurate in recalling the timing of past events. However, we found that age at smoking initiation was consistently reported up to two decades apart (see https://doi.org/10.1371/journal.pone.0201881.s001 and S3 Table).

The risk of relapse is the main issue when studying smoking cessation. About half of smokers attempts to quit every year, but less than 10% achieves abstinence for a period of at least 6 months.[40] The first year after a quit attempt is the period at highest risk for relapse.[17] In our study, age at cessation reported in a sub-sample of ex-smokers with follow-up data (ECRHS clinical) increased by two years on average after 20 years, probably indicating smoking relapses. However, we derived age at cessation from the last time point available, and over 90% of quitters had abstained from smoking for at least two years before the interview, suggesting that our findings mainly reflect smoking cessation over an extended period among subjects at a low risk of relapse. Moreover, the main results were confirmed in a sensitivity analysis using a more conservative definition of smoking cessation (having quitted for at least two years; S3 Fig).

### Conclusions

Our findings indicate that smoking cessation rates are increasing throughout Europe, especially for women around the age of 30, suggesting that there is a mounting awareness of the detrimental effects of smoking exposure. Scandinavian countries and the UK have greater cessation rates than the rest of Europe, where the implementation of tobacco control strategies should be intensified. Additional efforts should be made to prevent adolescents taking up smoking, as early initiation could make quitting smoking more challenging during later life.

## Supporting information

S1 Appendix Supplementary information on the original studies. (DOCX)

S1 Dataset Minimal dataset to replicate the analyses. The dataset includes: region (North, East, South, West Europe); sex (male = 1, female = 2), a = age in years, p = period (year of observation), c = cohort (birth year), d = number of quitters, py = person-years.
(CSV)

S1 Fig Study participants. (DOCX)

S2 Fig Distribution of ever smokers by study and year of interview. (DOCX)

S3 Fig Sensitivity analysis. Estimated trends in the rates (per 1,000/year) of smoking cessation (defined as having quitted smoking for at least two years) with 95% confidence intervals in females and males, by region. Trends were stratified by period: black lines refer to 1980–1989; red lines refer to 1990–1999, and green lines refer to 2000–2010.
(DOCX)

S1 Table Distribution of ever smokers included in the analysis (and total number of participants into the original studies with complete data) by region, centre and study. (DOCX)

S2 Table Cumulative incidence of smoking relapse by time since quitting. (DOCX)

S3 Table Comparison of the age at smoking initiation and cessation reported at different occasions in multi-wave studies. (DOCX)

## Abbreviations

ALEC: Ageing Lungs In European Cohorts
ECRHS: European Community Respiratory Health Survey
GA2LEN: Global Allergy and Asthma Network of Excellence
GEIRD: Gene Environment Interactions in Respiratory Diseases
IRR: Incidence Rate Ratio
ISAYA: Italian Study on Asthma in Young Adults
RHINE: Respiratory Health in Northern Europe

## References

[1] Carter MPH, Abnet CC, Feskanich D, Freedman ND, Hartge P, Lewis CE, et al Smoking and Mortality—Beyond Established Causes. N Eng J Med 2015;372:631–640. 10.1056/NEJMsa1407211 25671255

[2] World Health Organization (WHO) 2017. Report on the global tobacco epidemic, 2017. Monitoring tobacco use and prevention policies. Geneva: World Health Organization 2017. (http://apps.who.int/iris/bitstream/10665/255874/1/9789241512824-eng.pdf?ua=1&ua=1, accessed 1 October 2018)

[3] European Respiratory Society (ERS). The European Lung White Book. Chapter 9: Major risk factors—Tobacco Smoking. (https://www.erswhitebook.org/files/public/Chapters/09_tobacco_smoking.pdf, accessed 1 October 2018).

[4] Jha P, Ramasundarahettige C, Landsman V, Rostron B, Thun M, Anderson RN, et al 21st-century hazards of smoking and benefits of cessation in the United States. N Engl J Med 2013;368:341–350. 10.1056/NEJMsa1211128 23343063

[5] Eurobarometer. Special Report 458: Attitudes of Europeans toward tobacco and electronic cigarettes. May 2017. (https://ec.europa.eu/commfrontoffice/publicopinion/index.cfm/ResultDoc/download/DocumentKy/79002, accessed 1 October 2018)

[6] Marcon A, Pesce G, Calciano L, Bellisario V, Dharmage SC, Garcia-Aymerich J, et al Trends in smoking initiation in Europe over 40 years: a retrospective cohort study. PLoS One 2018;13(8):e0201881 10.1371/journal.pone.0201881 30133533 PMC6104979

[7] Jarvis D, Newson R, Lotvall J, Hastan D, Tomassen P, Keil T, et al Asthma in adults and its association with chronic rhinosinusitis: the GA2LEN survey in Europe. Allergy 2012;67:91–8. 10.1111/j.1398-9995.2011.02709.x 22050239

[8] de Marco R, Poli A, Ferrari M, Accordini S, Giammanco G, Bugiani M, et al The impact of climate and traffic-related NO2 on the prevalence of asthma and allergic rhinitis in Italy. Clin Exp Allergy 2002;32:1405–1412. 12372117 10.1046/j.1365-2745.2002.01466.x

[9] de Marco R, Accordini S, Antonicelli L, Bellia V, Bettin MD, Bombieri C, et al The Gene-Environment Interactions in Respiratory Diseases (GEIRD) project. Int Arch Allergy Immunol 2010;153(3):255–63. 10.1159/000283034 20150743

[10] Burney PG, Luczynska C, Chinn S, Jarvis D. The European Community Respiratory Health Survey. Eur Respir J 1994;7:954–60. 8050554 10.1183/09031936.94.07050954

[11] European Community Respiratory Health Survey II Steering Committee. The European Community Respiratory Health Survey II. Eur Respir J 2002;20:1071–9. 12449157 10.1183/09031936.02.00046802

[12] Amaral AFS, Newson RB, Abramson MJ, Antó JM, Bono R, Corsico AG, et al Changes in IgE sensitization and total IgE levels over a 20 years follow-up. J Allergy Clin Immunol 2016;137(6):1788–95. 10.1016/j.jaci.2015.09.037 26586040 PMC4889785

[13] de Marco R, Cappa V, Accordini S, Rava M, Antonicelli L, Bortolami O, et al Trends in the prevalence of asthma and allergic rhinitis in Italy between 1991 and 2010. Eur Respir J 2012;39:883–92. 10.1183/09031936.00061611 22005911

[14] Pesce G, Locatelli F, Cerveri I, Bugiani M, Pirina P, Johannessen A, et al Seventy years of asthma in Italy: age, period and cohort effects on incidence and remission of self-reported asthma from 1940 to 2010. PLoS One 2015;10(10):e0138570 10.1371/journal.pone.0138570 26439263 PMC4595078

[15] Toren K, Gislason T, Omenaas E, Jögi R, Forsberg B, Nyström L, et al A prospective study of asthma incidence and its predictors: the RHINE study. Eur Respir J 2004;24(6):942–6. 10.1183/09031936.04.00044804 15572536

[16] Bilano V, Gilmour S, Moffiet T, d'Espaignet ET, Stevens GA, Commar A, et al Global trends and projections for tobacco use, 1990–2025: an analysis of smoking indicators from the WHO Comprehensive Information Systems for Tobacco Control. Lancet 2015;385:966–76. 10.1016/S0140-6736(15)60264-1 25784347

[17] Garcia-Rodriguez O, Secades-Villa R, Florez-Salamanca L, Okuda M, Liu SM, Blanco C. Probability and predictors of relapse to smoking: results of the National Epidemiologic Survey on Alcohol and Related Conditions (NESARC). Drug Alcohol Depend 2013;132:479–85. 10.1016/j.drugalcdep.2013.03.008 23570817 PMC3723776

[18] Holm M, Schioler L, Andersson E, Forsberg B, Gislason T, Janson C, et al Predictors of smoking cessation: a longitudinal study in a large cohort of smokers. Respir Med 2017;123:164–9. 10.1016/j.rmed.2017.10.013 29229092

[19] Bogdanovica I, Godfrey F, McNeill A, Britton J. Smoking prevalence in the European Union: a comparison of national and transnational prevalence survey methods and results. Tob Control 2011;20:e4 10.1136/tc.2010.036103 20966129 PMC3003865

[20] Zatonski W, Przewoniak K Sulkowska U, West R, Wojtyla A. Tobacco smoking in countries of the European Union. Ann Agricul Environ Med 2012:19(2):181–192. 22742786

[21] Joossens L, Raw M. The Tobacco Control Scale: a new scale to measure country activity. Tobacco Control 2006;15:247–253. 10.1136/tc.2005.015347 16728757 PMC2564668

[22] Bosdriesz JR, Willemsen MC, Stronks K, Kunst AE. Tobacco control policy and socio-economic inequalities in smoking in 27 European countries. Drug Alcohol Dependence 2016;165:79–86 10.1016/j.drugalcdep.2016.05.020 27262899

[23] Jarvis MJ, Cohen JE, Delnevo CD, Giovino GA. Dispelling myths about gender differences in smoking cessation: population data from USA, Canada, and Britain. Tob Control 2013;22:356–60. 10.1136/tobaccocontrol-2011-050279 22649182

[24] Central Intelligence Agency (CIA). The World Factbook—Mother Mean Age at First Child. (https://www.cia.gov/library/publications/the-world-factbook/fields/2256.html, accessed 1 October 2018)

[25] Smedberg J, Lupattelli A, Mardby AC, Nordeng H. Characteristics of women who continue smoking during pregnancy: a cross-sectional study of pregnant women and new mothers in 15 European countries. BMC Pregnancy Childbirth 2014;14:213 10.1186/1471-2393-14-213 24964728 PMC4080751

[26] Tsakiridis I, Mamopoulos A, Papazisis G, Petousis S, Liozidou A, Athanasiadis A, et al Prevalence of smoking during pregnancy and associated risk factors: a cross-sectional study in Northern Greece. Eur J Pub Health 2018;28(2):321–5. 29342261 10.1093/eurpub/cky004

[27] Einarson A, Riordan S. Smoking in pregnancy and lactation: a review of risks and cessation strategies. Eur J Clin Pharmacol 2009;65:325–30. 10.1007/s00228-008-0609-0 19169678

[28] Wigginton B, Lee C. Stigma and hostility towards pregnant smokers: does individuating information reduce the effect? Psychol Health 2013;28(8):862–73 10.1080/08870446.2012.762101 23343130

[29] McBride CM, Baucom DH, Peterson BL, Pollak KI, Palmer C, Westman E, et al Prenatal and postpartum smoking abstinence: a partner-assisted approach. Am J Prev Med 2004;27(3):232–8. 10.1016/j.amepre.2004.06.005 15450636

[30] Janson C, Kunzli N, de Marco R, Chinn S, Jarvis D, Svanes C, et al Changes in active and passive smoking in the European Community Respiratory Health Survey. Eur Respir J 2006;27:517–24. 10.1183/09031936.06.00106605 16507851

[31] Flemming K, Graham H, McCaughan D, Angus K, Bauld L. The barriers and facilitators to smoking cessation experienced by women’s partners during pregnancy and the post-partum: a systematic review of qualitative research. BMC Public Health 2015;15:849 10.1186/s12889-015-2163-x 26335935 PMC4558795

[32] Coambs RB, Li S, Kozlowski LT. Age interacts with heaviness of smoking in predicting success in cessation of smoking. Am J Epidemiol 1992;135:240–6. 1546699 10.1093/oxfordjournals.aje.a116277

[33] Choi SH, Stommel M. Impact of age at smoking initiation on smoking-related morbidity and all-cause mortality. Am J Prev Med 2017;53(1):33–41. 10.1016/j.amepre.2016.12.009 28169018

[34] Accordini S, Calciano L, Johannessen A, Portas L, Benediktsdóttir B, Bertelsen RJ, et al A three-generation study of tobacco smoking with asthma. Int J Epidemiol 2018;47(4):1106–1117. 10.1093/ije/dyy031 29534228 PMC6124624

[35] Breslau N, Peterson EL. Smoking cessation in young adults: age at initiation of cigarette smoking and other suspected influences. Am J Pub Health 1996;86(2):214–20. 8633738 10.2105/ajph.86.2.214 PMC1380330

[36] Chen J, Millar WJ. Age at smoking initiation: implications for quitting. Health Rep 1998;9(4):39–46. 9836879

[37] Kocak ND, Eren A, Boga S, Aktürk ÜA, Öztürk ÜA, Arınç S, et al Relapse rate and factors related to relapse in a 1-year follow-up of subjects participating in a smoking cessation program. Respir Care 2015;60(12):1796–803. 10.4187/respcare.03883 26286738

[38] Breslau N, Fenn N, Peterson EL. Early smoking initiation and nicotine dependence in a cohort of young adults. Drug Alcohol Dependance 1993;33:129–37. 10.1016/0376-8716(93)90054-t 8261877

[39] Olivieri M, Poli A, Zuccaro P, Ferrari M, Lampronti G, de Marco R, et al Tobacco smoke exposure and serum cotinine in a random sample of adults living in Verona, Italy. Arch Environ Health 2002;57:355–9. 10.1080/00039890209601421 12530604

[40] Babb S, Malarcher A, Schauer G, Asman K, Jamal A. Quitting smoking among adults—United States, 2000–2015. MMWR Morb Mortal Wkly Rep 2017;65(52):1457–64. 10.15585/mmwr.mm6552a1 28056007

