# Supplementary material for: Time and age trends in smoking cessation in Europe
Source: PLoS One. 2019 Feb 7;14(2):e0211976. doi: 10.1371/journal.pone.0211976 (PMC6366773; doi:10.1371/journal.pone.0211976)
Supplement: S1 Appendix — (DOCX) [file pone.0211976.s001.docx]

# S1 Appendix. Supplementary information on the original studies

***European Community Respiratory Health Survey (ECRHS)***

*Co-ordinating centre*: P. Burney, S. Chinn, C. Luczynska†, D. Jarvis, E. Lai (London).

*Project management group*: P. Burney (Project leader-UK), S. Chinn (UK), C. Luczynska† (UK), D. Jarvis (UK), P. Vermeire† (Antwerp), H. Kesteloot (Leuven), J. Bousquet (Montpellier), D. Nowak (Hamburg), J. Prichard† (Dublin), R. de Marco† (Verona), B. Rijcken (Groningen), J.M. Anto (Barcelona), J. Alves (Oporto), G. Boman (Uppsala), N. Nielsen (Copenhagen), P. Paoletti (Pisa).

*Steering Committee of ECRHS II*: U. Ackermann-Liebrich (University of Basel, Switzerland); N. Kuenzli (University of Basel, and University of Southern California, Los Angeles, USA); J.M. Antó and J. Sunyer (Institut Municipal d’Investigació Médica (IMIM-IMAS), Universitat Pompeu Fabra (UPF), Spain); P. Burney (project leader), S Chinn, D. Jarvis, J. Knox and C. Luczynska† (King’s College London, UK); I. Cerveri (University of Pavia, Italy); R. de Marco† (University of Verona, Italy); T. Gislason (Iceland University Hospital, Iceland); J. Heinrich and M. Wjst (GSF–Institute of Epidemiology, Germany); C. Janson (Uppsala University, Sweden); B. Leynaert and F. Neukirch (Institut National de la Sante´ et de la Recherche Medicale (INSERM), France); J. Schouten (University of Groningen, The Netherlands); C. Svanes (University of Bergen, Norway); P. Vermeire† (University of Antwerp, Belgium).

*Principal investigators and senior scientific teams of ECRHS II*: Australia: (M. Abramson, E.H Walters, J. Raven); Belgium: South Antwerp and Antwerp City (P. Vermeire†, J. Weyler, M. van Sprundel, V. Nelen); Estonia: Tartu (R. Jõgi, A. Soon); France: Paris (F. Neukirch, B. Leynaert, R. Liard, M. Zureik), Grenoble (I. Pin, J. Ferran-Quentin); Bordeaux (A. Taytard, C. Raherison), Montpellier (J. bBousquet, P.J. Bousquet); Germany: Erfurt (J. Heinrich, M. Wjst, C. Frye, I. Meyer); Iceland: Reykjavik (T. Gislason, E. Bjornsson, D. Gislason, K.B. Jörundsdóttir); Italy: Turin (R. Bono, M. Bugiani, P.Piccioni, E. Caria, A. Carosso, E. Migliore, G. Castiglioni), Verona (R. de Marco†, G. Verlato, E. Zanolin, S. Accordini, A. Poli, V. Lo Cascio, M. Ferrari, I. Cazzoletti), Pavia (A. Marinoni, S. Villani, M. Ponzio, F. Frigerio, M. Comelli, M. Grassi, I. Cerveri, A. Corsico); The Netherlands: Groningen and Geleen (J. Schouten, M. Kerkhof); Norway: Bergen (A. Gulsvik, E. Omenaas, C. Svanes, B. Laerum); Spain: Albacete (J. Martinez-Moratalla Rovira, E. Almar, M. Arévalo, C. Boix, G. González, J.M. Ignacio García, J. Solera, J. Damián), Galdakao (N. Muñozguren, J. Ramos, I. Urrutia, U. Aguirre), Barcelona (J.M. Antó, J. Sunyer, M. Kogevinas, J.P. Zock, X. Basagaña, A. Jaen, F. Burgos, C. Acosta), Huelva (J. Maldonado, A. Pereira, J.L. Sanchez), Oviedo (F. Payo, I. Huerta, A. de la Vega, L. Palenciano, J. Azofra, A. Cañada); Sweden: Göteborg (K. Toren, L. Lillienberg, A.C. Olin, B. Balder, A. Pfeifer-Nilsson, R. Sundberg), Umea (E. Norrman, M. Soderberg, K.A. Franklin, B. Lundback, B. Forsberg, L. Nystrom), Uppsala (C. Janson, G. Boman, D. Norback, G. Wieslander, M. Gunnbjornsdottir); Switzerland: Basel (N. Küenzli, B. Dibbert, M. Hazenkamp, M. Brutsche, U. Ackermann-Liebrich); United Kingdom: Ipswich (D. Jarvis, R. Hall, D. Seaton), Norwich (D. Jarvis, B. Harrison).

*Principal Investigators and senior scientific teams of ECRHS III*: Australia: Melbourne (M. Abramson, G. Benke, S. Dharmage, B. Thompson, S. Kaushik, M. Matheson); Belgium: South Antwerp & Antwerp City (J. Weyler, H. Bentouhami, V. Nelen); Denmark: Aarhus (V. Schlunssen, T. Sigsgaard, R.Dahl); Estonia: Tartu (R. Jõgi, H.Orru); France: Bordeaux (C. Raherison, P.O. Girodet), Grenoble (I. Pin, V. Siroux, J. Ferran, J.L. Cracowski), Montpellier (P. Demoly, A.Bourdin, I. Vachier), Paris (B. Leynaert, D. Soussan, D. Courbon, C. Neukirch, L. Alavoine, X. Duval, I. Poirier); Germany: Erfurt (J. Heinrich, E. Becker, G. Woelke, O. Manuwald), Hamburg (H. Magnussen, D. Nowak, A.M. Kirsten); Iceland: Reykjavik (T. Gislason, B. Benediktsdottir, D. Gislason, E.S. Arnardottir, M. Clausen, G. Gudmundsson, L. Gudmundsdottir, H. Palsdottir, K. Olafsdottir, S. Sigmundsdottir, K. Bara-Jörundsdottir); Italy: Pavia (I. Cerveri, A.Corsico, A. Grosso, F. Albicini, E. Gini, E.M. Di Vincenzo, V. Ronzoni, S. Villani, F. Campanella, M. Gnesi, F. Manzoni, L. Rossi, O. Ferraro),Turin: (M. Bugiani, R. Bono, P. Piccioni, R. Tassinari, V. Bellisario, G. Trucco), Verona: (R de Marco†, S. Accordini, L. Calciano, L. Cazzoletti, M. Ferrari, A.M. Fratta Pasini, F. Locatelli, P. Marchetti, A. Marcon, E. Montoli, G. Nguyen, M. Olivieri, C. Papadopoulou, C.Posenato, G. Pesce, P. Vallerio, G. Verlato, E. Zanolin); Netherlands: Groningen (H.M. Boezen); Norway: (C. Svanes, E. Omenaas, A. Johannessen, T. Skorge, F. Gomez Real); Spain: Albacete (J. Martinez-Moratalla Rovira, E. Almar, A. Mateos, S. García, A. Núñez, P. López, R. Sánchez, E. Mancebo), Barcelona:(J.M. Antó, J.P. Zock, J. Garcia-Aymerich, M. Kogevinas, X. Basagaña, A.E. Carsin, F. Burgos, C. Sanjuas, S. Guerra, B. Jacquemin, P. Davdand); Galdakao (N. Muñozguren, I. Urrutia, U. Aguirre, S. Pascual); Huelva (J. Antonio Maldonado, A. Pereira, J. Luis Sánchez, L. Palacios); Oviedo (F. Payo, I. Huerta, N. Sánchez, M. Fernández, B. Robles); Sweden: Göteborg (K. Torén, M. Holm, J.L. Kim, A.C. Olin, A. Dahlman-Höglund), Umea (B. Forsberg, L. Braback, L. Modig, B. Järvholm, H. Bertilsson, K.A. Franklin, C. Wahlgreen), Uppsala:(B. Andersson, D. Norback, U. Spetz Nystrom, G. Wieslander, G.M. Bodinaa Lund, K Nisser); Switzerland: Basel (N.M. Probst-Hensch, N. Künzli, D. Stolz, C. Schindler, T. Rochat, J.M. Gaspoz, E. Zemp Stutz, M. Adam, C. Autenrieth, I. Curjuric, J. Dratva, A. Di Pasquale, R. Ducret-Stich, E. Fischer, L. Grize, A. Hensel, D. Keidel, A. Kumar, M. Imboden, N. Maire, A. Mehta, H. Phuleria, M. Ragettli, M. Ritter, E. Schaffner, G.A. Thun, A. Ineichen, T. Schikowski, M. Tarantino, M. Tsai); UK: London (P. Burney, D. Jarvis, S. Kapur, R. Newson, J. Potts), Ipswich (N. Innes), Norwich (A. Wilson).

*Financial support for ECRHS I*: The co-ordination of this work was supported by the European Commission and the authors and participants are grateful to the late C. Baya and M. Hallen for their help during the study and K. Vuylsteek and the members of the COMAC for their support. The following grants helped to fund the local studies: Belgium: Belgian Science Policy Office, National Fund for Scientific Research; Estonia: Estonian Science Foundation, grant no 1088; France: Ministère de la Santé, Glaxo France, Institut Pneumologique d'Aquitaine, Contrat de Plan Etat-Région Languedoc-Rousillon, CNMATS, CNMRT (90MR/10, 91AF/6), Ministre delegué de la santé, RNSP, France, GSF; Germany: Bundesminister für Forschung und Technologie; Italy: Ministero dell'Università e della Ricerca Scientifica e Tecnologica, CNR, Regione Veneto grant RSF n. 381/05.93; Norway: Norwegian Research Council project no. 101422/310; Portugal: Glaxo Farmacêutica Lda, Sandoz Portugesa; Spain: Fondo de Investigación Sanitaria (#91/0016-060-05/E, 92/0319 and #93/0393), Hospital General de Albacete, Hospital General Juan Ramón Jiménez, Dirección Regional de Salud Pública (Consejería de Sanidad del Principado de Asturias), CIRIT (1997 SGR 00079) and Servicio Andaluz de Salud; Sweden: The Swedish Medical Research Council, the Swedish Heart Lung Foundation, the Swedish Association against Asthma and Allergy; Switzerland: Swiss National Science Foundation grant 4026-28099; UK: National Asthma Campaign, British Lung Foundation, Department of Health, South Thames Regional Health Authority.

*Financial Support for ECRHS II***:** Belgium: Antwerp: Fund for Scientific Research (grant code, G.0402.00), University of Antwerp, Flemish Health Ministry; Estonia: Tartu Estonian Science Foundation grant no 4350, France: (All) Programme Hospitalier de Recherche Clinique—Direction de la Recherche Clinique (DRC) de Grenoble 2000 number 2610, Ministry of Health, Ministère de l’Emploi et de la Solidarité, Direction Génerale de la Santé, Centre Hospitalier Universitaire (CHU) de Grenoble; Bordeaux: Institut Pneumologique d’Aquitaine; Grenoble: Comite des Maladies Respiratoires de l’Isere Montpellier: Aventis (France), Direction Regionale des Affaires Sanitaires et Sociales Languedoc-Roussillon; Paris: Union Chimique Belge-Pharma (France), Aventis (France), Glaxo France, Germany: Erfurt: GSF—National Research Centre for Environment and Health, Deutsche Forschungsgemeinschaft (grant code, FR1526/1-1); Hamburg: GSF—National Research Centre for Environment and Health, Deutsche Forschungsgemeinschaft (grant code, MA 711/4-1); Iceland: Reykjavik, Icelandic Research Council, Icelandic University Hospital Fund; Italy: Pavia: GlaxoSmithKline Italy, Italian Ministry of University and Scientific and Technological Research (MURST), Local University Funding for Research 1998 and 1999; Turin: Azienda Sanitaria Locale 4 Regione Piemonte (Italy), Azienda Ospedaliera Centro Traumatologico Ospedaliero/Centro Traumatologico Ortopedico—Istituto Clinico Ortopedico Regina Maria Adelaide Regione Piemonte; Verona: Ministero dell’Università e della Ricerca Scientifica (MURST), Glaxo Wellcome s.p.a.; Norway: Bergen: Norwegian Research Council, Norwegian Asthma and Allergy Association, Glaxo Wellcome AS, Norway Research Fund; Spain: Fondo de Investigacion Santarias (grant codes, 97/0035-01, 99/0034-01 and 99/0034-02), Hospital Universitario de Albacete, Consejeria de Sanidad; Barcelona: Sociedad Espanola de Neumología y Cirugía Toracica, Public Health Service (grant code, R01 HL62633-01), Fondo de Investigaciones Santarias (grant codes, 97/0035-01, 99/0034-01, and 99/0034-02), Consell Interdepartamentalde Recerca i Innovació Tecnológica (grant code, 1999SGR 00241) Instituto de Salud Carlos III; Red de Centros de Epidemiología y Salud Pública, C03/09,Red de Bases moleculares y fisiológicas de las Enfermedades Respiratorias, C03/011and Red de Grupos Infancia y Medio Ambiente G03/176; Huelva: Fondo de Investigaciones Santarias (grant codes, 97/0035-01, 99/0034-01, and 99/0034-02); Galdakao: Basque Health Department; Oviedo: Fondo de Investigaciones Sanitaria (97/0035-02, 97/0035, 99/0034-01, 99/0034-02, 99/0034-04, 99/0034-06, 99/350, 99/0034--07), European Commission (EU-PEAL PL01237), Generalitat de Catalunya (CIRIT 1999 SGR 00214), Hospital Universitario de Albacete, Sociedad Española de Neumología y Cirugía Torácica (SEPAR R01 HL62633-01) Red de Centros de Epidemiología y Salud Pública (C03/09), Red de Bases moleculares y fisiológicas de las Enfermedades Respiratorias (C03/011) and Red de Grupos Infancia y Medio Ambiente (G03/176; 97/0035-01, 99/0034-01, and 99/0034-02); Sweden: Göteborg, Umea, Uppsala: Swedish Heart Lung Foundation, Swedish Foundation for Health Care Sciences and Allergy Research, Swedish Asthma and Allergy Foundation, Swedish Cancer and Allergy Foundation, Swedish Council for Working Life and Social Research (FAS); Switzerland: Basel Swiss National Science Foundation, Swiss Federal Office for Education and Science, Swiss National Accident Insurance Fund; UK: Ipswich and Norwich: Asthma UK (formerly known as National Asthma Campaign).

*Financial Support for ECRHS III*: Belgium: Antwerp South and Antwerp City: Research Foundation Flanders (FWO), grant code G.0.410.08.N.10; Estonia: Tartu: SF0180060s09 from the Estonian Ministry of Education; France: (All) Ministère de la Santé, Programme Hospitalier de Recherche Clinique (PHRC) national 2010, Bordeaux: INSERM U897 Université Bordeaux segalen, Grenoble: Comite Scientifique AGIRadom 2011, Paris: Agence Nationale de la Santé, Région Ile de France, domaine d’intérêt majeur (DIM); Germany: Erfurt: German Research Foundation HE 3294/10-1, Hamburg: German Research Foundation MA 711/6-1, NO 262/7-1; Iceland: Reykjavik: The Landspitali University Hospital Research Fund, University of Iceland Research Fund, ResMed Foundation, California, USA, Orkuveita Reykjavikur (Geothermal plant), Vegagerðin (The Icelandic Road Administration (ICERA); Italy: All Italian centres were funded by the Italian Ministry of Health, Chiesi Farmaceutici SpA, in addition Verona was funded by Cariverona foundation, Education Ministry (MIUR); Norway: Norwegian Research council grant no 214123, Western Norway Regional Health Authorities grant no 911631, Bergen Medical Research Foundation; Spain: Fondo de Investigación Sanitaria (PS09/02457, PS09/00716, PS09/01511, PS09/02185, PS09/03190), Servicio Andaluz de Salud, Sociedad Española de Neumología y Cirurgía Torácica (SEPAR 1001/2010), Fondo de Investigación Sanitaria (PS09/02457), Barcelona: Fondo de Investigación Sanitaria (FIS PS09/00716), Galdakao: Fondo de Investigación Sanitaria (FIS 09/01511), Huelva: Fondo de Investigación Sanitaria (FIS PS09/02185) and Servicio Andaluz de Salud, Oviedo: Fondo de Investigación Sanitaria (FIS PS09/03190); Sweden: All centres were funded by The Swedish Heart and Lung Foundation, The Swedish Asthma and Allergy Association, The Swedish Association against Lung and Heart Disease, Swedish Research Council for health, working life and welfare (FORTE), Göteborg: also received further funding from the Swedish Council for Working life and Social Research. Umea: also received funding from Vasterbotten Country Council ALF grant; Switzerland: The Swiss National Science Foundation (grants no 33CSCO-134276/1, 33CSCO-108796, 3247BO-104283, 3247BO-104288, 3247BO-104284, 3247-065896, 3100-059302, 3200-052720, 3200-042532, 4026-028099), The Federal office for forest, environment and landscape, The Federal Office of Public Health, The Federal Office of Roads and Transport, the canton’s government of Aargan, Basel-Stadt, Basel-Land, Geneva, Luzern, Ticino, Valais and Zürich, the Swiss Lung League, the canton's Lung League of Basel Stadt/ Basel, Landschaft, Geneva, Ticino, Valais and Zurich, SUVA, Freiwillige Akademische Gesellschaft, UBS Wealth Foundation, Talecris Biotherapeutics GmbH, Abbott Diagnostics, European Commission 018996 (GABRIEL), Wellcome Trust WT 084703MA; UK: Medical Research Council (Grant Number 92091), support also provided by the National Institute for Health Research through the Primary Care Research Network.

***Respiratory Health in Northern Europe (RHINE) study***

*Project leader*: C. Janson.

*Principal investigators*: Reykjavik: T. Gislason; Bergen: C. Svanes; Aarhus: V. Schlünssen; Tartu: R. Jõgi; Gothenburg: M. Holm; Umeå: B. Forsberg; Uppsala: C. Janson.

*Financial Support***:** the study was funded by the Swedish Heart and Lung Foundation, the Swedish Association Against Asthma and Allergy, the Swedish Association against Heart and Lung Disease, the Swedish Council for Working Life and Social Research, the Bror Hjerpstedt Foundation, the Vårdal Foundation for Health Care and Allergic Research, The Faculty of Health, Aarhus University, Denmark (Project No. 240008), The Wood Dust Foundation (Project No. 444508795), The Danish Lung Association, The Norwegian Research Council project 135773/330, The Norwegian Asthma and Allergy Association, The Icelandic Research Council and the Estonian Science Foundation (Grant No. 4350). Vivi Schlünssen, Thorarinn Gislason and Cecilie Svanes are members of the COST BM1201 network.

***Global Allergy and Asthma Network of Excellence (GA^2^LEN) study***

*GA^2^LEN Project leader:* T. Zuberbier.

*Project leader for Epidemiological Surveys*: P. Burney.

*Financial Support***:** the collection of epidemiological data used in this analysis was funded through the Sixth European Union Framework program for research, contract no. FOOD-CT-2004-506378.

***Italian Study on Asthma in Young Adults (ISAYA)***

*Project leader***:** R. de Marco†.

*Financial Support***:** the study was supported by the Italian Ministry of University and Scientific and Technological Research; GlaxoSmithKline Italia; National Health Service, AUSL Ferrara; National Health Service, AUSL Pavia; National Health Service, ASL 4 Turin; IRCCS San Matteo; Municipality of Udine; Councillorship for Health, Modena Province; Pavia Province; Siracusa Province; and the Veneto Region.

***Gene Environment Interactions in Respiratory Diseases study (GEIRD)***

*Project leader*: R. de Marco†.

*Co-ordinating centre*: R. de Marco†, G. Verlato, M. E. Zanolin, S. Accordini, L. Cazzoletti, A. Marcon (Verona).

*Steering Committee*: L. Antonicelli, C. Bombieri, M. Bugiani, L. Casali, I. Cerveri, L. Cominacini, R. de Marco†, M. Ferrari, M Olivieri, L. Perbellini, P. Pirina, A. Poli, G. Rolla, G. Verlato, S. Villani, M. E. Zanolin.

*Financial Support***:** the study was funded by the Cariverona Foundation (Verona, Italy); the Italian Ministry of Health (Ricerca finalizzata 2009); Chiesi Farmaceutici S.p.A.; and the Agenzia Italiana del Farmaco (AIFA).

† deceased
