# Supplementary material for: Time and age trends in smoking cessation in Europe
Source: PLoS One. 2019 Feb 7;14(2):e0211976. doi: 10.1371/journal.pone.0211976 (PMC6366773; doi:10.1371/journal.pone.0211976)
Supplement: S1 Fig — (DOCX) [file pone.0211976.s003.docx]

**S1 Fig. Study participants**


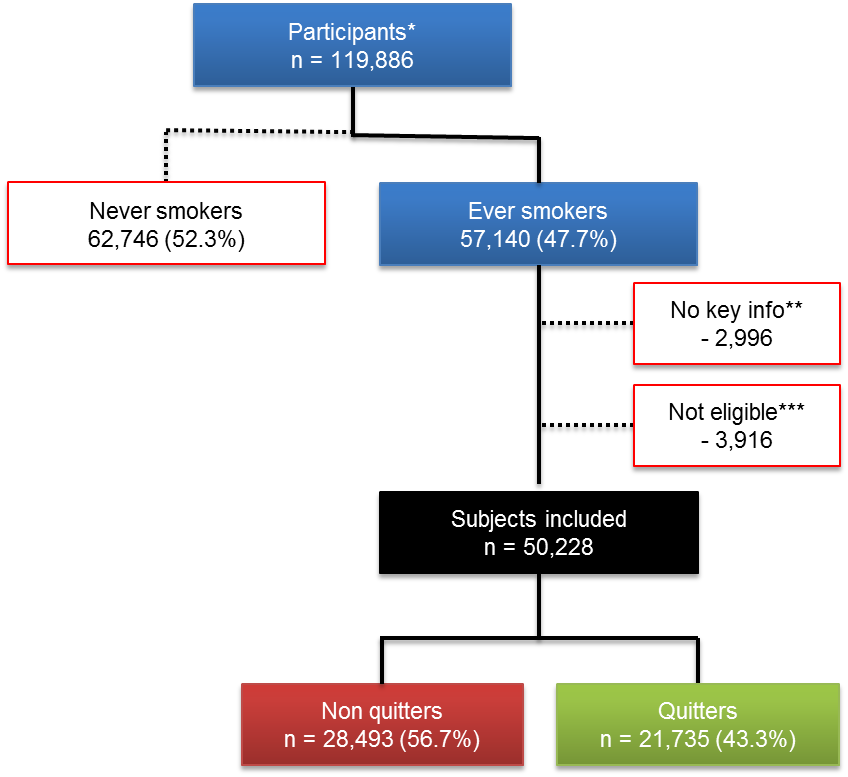


* subjects with complete information on sex, year of birth, and smoking status

** subjects with missing data on age at smoking initiation and/or age at cessation if ex-smoker

*** subjects who quit smoking before the age of 16 (n=117) or before 1980 (n=3,777); subjects who started smoking at age ≥60 years (n=4) or after 2010 (n=2); subjects aged <16 years when surveyed (n=13); or subjects aged ≥60 years in 1980 (n=3).
