# Supplementary material for: Time and age trends in smoking cessation in Europe
Source: PLoS One. 2019 Feb 7;14(2):e0211976. doi: 10.1371/journal.pone.0211976 (PMC6366773; doi:10.1371/journal.pone.0211976)
Supplement: S2 Fig — (DOCX) [file pone.0211976.s004.docx]

**S2 Fig. Distribution of ever smokers by study and year of interview**

| Year  Study | 1991 | 1992 | 1993 | 1994 | | 1995 | | 1996 | | 1997 | | 1998 | | 1999 | 2000 | 2001 | 2002 | | 2003 | | 2004 | | 2005 | | 2006 | | 2007 | | 2008 | | 2009 | | 2010 | 2011 | 2012 | 2013 | |
| --- | --- | --- | --- | --- | --- | --- | --- | --- | --- | --- | --- | --- | --- | --- | --- | --- | --- | --- | --- | --- | --- | --- | --- | --- | --- | --- | --- | --- | --- | --- | --- | --- | --- | --- | --- | --- | --- |
| ECRHS clinical | **7,630** | | | |  | |  | |  | |  | | 4,073 | | | | |  | |  | |  | |  | |  | |  | |  | | 4,108 | | | | |  |
| RHINE |  |  |  |  | |  | |  | |  | |  | |  |  |  |  | |  | |  | |  | |  | |  | |  | |  | | 4,029 | | |  | |
| ECRHS-Italy |  |  |  |  | |  | |  | |  | | **1,808** | | | | |  | |  | |  | |  | |  | |  | | 653 | | | |  |  |  |  | |
| ISAYA |  |  |  |  | |  | |  | |  | | **8,788** | | | |  |  | |  | |  | |  | |  | |  | | 1,101 | | | |  |  |  |  | |
| GEIRD |  |  |  |  | |  | |  | |  | |  | |  |  |  |  | |  | |  | | **5,873** | | | | | | | | | | |  |  |  | |
| GA^2^LEN |  |  |  |  | |  | |  | |  | |  | |  |  |  |  | |  | |  | |  | |  | | **22,100** | | | | | |  |  |  |  | |

Grey boxes indicate subjects identified either in cross-sectional studies or at the first contact of cohort studies; white boxes indicate follow-up data
