# Supplementary material for: Time and age trends in smoking cessation in Europe
Source: PLoS One. 2019 Feb 7;14(2):e0211976. doi: 10.1371/journal.pone.0211976 (PMC6366773; doi:10.1371/journal.pone.0211976)
Supplement: S3 Fig — Trends were stratified by period: black lines refer to 1980–1989; red lines refer to 1990–1999, and green lines refer to 2000–2010. (DOCX) [file pone.0211976.s005.docx]

**S3 Fig. Sensitivity analysis. Estimated trends in the rates (per 1,000/year) of smoking cessation (defined as having quitted smoking for at least two years) with 95% confidence intervals in females and males, by region.**

| **** |
| --- |
| **** |

Trends were stratified by period: black lines refer to 1980-1989; red lines refer to 1990-1999, and green lines refer to 2000-2010.
