# Supplementary material for: Time and age trends in smoking cessation in Europe
Source: PLoS One. 2019 Feb 7;14(2):e0211976. doi: 10.1371/journal.pone.0211976 (PMC6366773; doi:10.1371/journal.pone.0211976)
Supplement: S1 Table — (DOCX) [file pone.0211976.s006.docx]

**S1 Table. Distribution of ever smokers included in the analysis (and total number of participants into the original studies with complete data) by region, centre and study**

| **REGION**  **Country**  Centre | **ECRHS**  **clinical** | **RHINE** | **ECRHS-Italy** | **GA^2^LEN** | **ISAYA** | **GEIRD** | **TOTAL** |
| --- | --- | --- | --- | --- | --- | --- | --- |
| **NORTH**  **EUROPE** |  |  |  |  |  |  | **18,255 (50,890)** |
| **Denmark** |  |  |  |  |  |  |  |
| Aarhus | 201 (382) | 791 (1,964) |  |  |  |  | 992 (2,346) |
| Odense |  |  |  | 1,517 (3,370) |  |  | 1,517 (3,370) |
| **Finland** |  |  |  |  |  |  |  |
| Helsinki |  |  |  | 802 (1,833) |  |  | 802 (1,833) |
| **Iceland** |  |  |  |  |  |  |  |
| Reykjavik | 324 (563) | 610 (1,461) |  |  |  |  | 934 (2,024) |
| **Norway** |  |  |  |  |  |  |  |
| Bergen | 458 (871) | 631 (1,548) |  |  |  |  | 1,089 (2,419) |
| Oslo |  |  |  | 63 (1,527) |  |  | 63 (1,527) |
| **Sweden** |  |  |  |  |  |  |  |
| Gothenburg | 361 (682) | 553 (1,212) |  | 2,904 (8,487) |  |  | 3,818 (10,381) |
| Stockholm |  |  |  | 2,217 (5,882) |  |  | 2,217 (5,882) |
| Umea | 223 (552) | 549 (1,534) |  | 1,595 (6,129) |  |  | 2,367 (8,215) |
| Uppsala | 280 (622) | 531 (1,467) |  | 1,876 (6,176) |  |  | 2,687 (8,265) |
| **United**  **Kingdom** |  |  |  |  |  |  |  |
| Caerphilly | 161 (376) |  |  |  |  |  | 161 (376) |
| Ipswich | 178 (448) |  |  |  |  |  | 178 (448) |
| London |  |  |  | 698 (1,989) |  |  | 698 (1,989) |
| Norwich | 203 (473) |  |  |  |  |  | 203 (473) |
| Southampton |  |  |  | 529 (1,342) |  |  | 529 (1,342) |
| **EAST**  **EUROPE** |  |  |  |  |  |  | **5,013 (10,587)** |
| **Estonia** |  |  |  |  |  |  |  |
| Tartu | 214 (414) | 364 (902) |  |  |  |  | 578 (1,316) |
| **Macedonia** |  |  |  |  |  |  |  |
| Skopje |  |  |  | 1,723 (3,618) |  |  | 1,723 (3,618) |
| **Poland** |  |  |  |  |  |  |  |
| Katowice |  |  |  | 1,314 (2,645) |  |  | 1,314 (2,645) |
| Krakow |  |  |  | 561 (1,262) |  |  | 561 (1,262) |
| Lodz |  |  |  | 837 (1,746) |  |  | 837 (1,746) |
| **SOUTH**  **EUROPE** |  |  |  |  |  |  | **19,179 (42,094)** |
| **Italy** |  |  |  |  |  |  |  |
| Ancona |  |  |  |  |  | 752 (1,856) | 752 (1,856) |
| Ferrara |  |  |  |  | 959 (2,106) |  | 959 (2,106) |
| Palermo |  |  |  | 340 (985) |  |  | 340 (985) |
| Pavia | 174 (310) |  | 356 (701) |  | 1,286 (2,444) | 622 (1,420) | 2,438 (4,875) |
| Pisa |  |  |  |  | 1,093 (2,408) |  | 1,093 (2,408) |
| Salerno |  |  |  |  |  | 695 (1,777) | 695 (1,777) |
| Sassari |  |  |  |  | 1,011 (2,052) | 1021 (2,201) | 2,032 (4,253) |
| Sassuolo |  |  |  |  | 1051 (2130) |  | 1051 (2130) |
| Siracusa |  |  |  |  | 348 (1,184) |  | 348 (1,184) |
| Terni |  |  |  |  |  | 745 (1,640) | 745 (1,640) |
| Torino | 130 (244) |  | 625 (1,266) |  | 1,080 (2,264) | 744 (1,663) | 2,579 (5,437) |
| Udine |  |  |  |  | 872 (2,081) |  | 872 (2,081) |
| Verona | 182 (342) |  | 827 (1,737) |  | 1,088 (2,160) | 1,294 (2,985) | 3,391 (7,224) |
| **Portugal** |  |  |  |  |  |  |  |
| Coimbra |  |  |  | 606 (2,201) |  |  | 606 (2,201) |
| **Spain** |  |  |  |  |  |  |  |
| Albacete | 285 (435) |  |  |  |  |  | 285 (435) |
| Barcelona | 247 (390) |  |  |  |  |  | 247 (390) |
| Galdakao | 317 (486) |  |  |  |  |  | 317 (486) |
| Huelva | 179 (270) |  |  |  |  |  | 179 (270) |
| Oviedo | 250 (356) |  |  |  |  |  | 250 (356) |
| **WEST**  **EUROPE** |  |  |  |  |  |  | **7,781 (16,315)** |
| **Belgium** |  |  |  |  |  |  |  |
| Antwerp City | 331 (564) |  |  |  |  |  | 331 (564) |
| Antwerp South | 269 (558) |  |  |  |  |  | 269 (558) |
| Ghent |  |  |  | 808 (1,861) |  |  | 808 (1,861) |
| **France** |  |  |  |  |  |  |  |
| Grenoble | 248 (474) |  |  |  |  |  | 248 (474) |
| Montpellier |  |  |  | 460 (1,371) |  |  | 460 (1,371) |
| **Germany** |  |  |  |  |  |  |  |
| Brandenburg |  |  |  | 1,043 (2,258) |  |  | 1,043 (2,258) |
| Erfurt | 457 (731) |  |  |  |  |  | 457 (731) |
| Hamburg | 776 (1,252) |  |  |  |  |  | 776 (1,252) |
| Munich |  |  |  | 904 (2,025) |  |  | 904 (2,025) |
| **The**  **Netherlands** |  |  |  |  |  |  |  |
| Amsterdam |  |  |  | 1,303 (3,147) |  |  | 1,303 (3,147) |
| Bergen op Zoom | 285 (451) |  |  |  |  |  | 285 (451) |
| Geleen | 219 (395) |  |  |  |  |  | 219 (395) |
| Groningen | 228 (378) |  |  |  |  |  | 228 (378) |
| **Switzerland** |  |  |  |  |  |  |  |
| Basel | 450 (850) |  |  |  |  |  | 450 (850) |
| **TOTAL** | **7,630 (13,869)** | **4,029 (10,088)** | **1,808 (3,704)** | **22,100 (59,854)** | **8,788 (18,829)** | **5,873 (13,542)** | **50,228 (119,886)** |
