# Supplementary material for: Time and age trends in smoking cessation in Europe
Source: PLoS One. 2019 Feb 7;14(2):e0211976. doi: 10.1371/journal.pone.0211976 (PMC6366773; doi:10.1371/journal.pone.0211976)
Supplement: S2 Table — (DOCX) [file pone.0211976.s007.docx]

**S2 Table. Cumulative incidence of smoking relapse by time since quitting***

|  | follow-up time (years)  mean (SD) | active smokers at last follow-up/ex-smokers at baseline n/N (%) | | |
| --- | --- | --- | --- | --- |
|  |  | Overall | Quit since  < 2 years | Quit since  ≥2 years |
| ECRHS clinical | 17.7 (4.2) | 228/1954 (11.7%) | 75/271 (27.7%) | 153/1683 (9.1%) |
| ISAYA | 9.8 (0.6) | 54/282 (15.2%) | 15/46 (32.6%) | 39/309 (12.6%) |
| Overall | 16.5 (4.8) | 282/2309 (12.2%) | 90/317 (28.4%) | 192/1992 (9.6%) |

* Calculated among ex-smokers at the baseline interview of the studies with follow-up data.

This table reports the cumulative incidence of relapse among subjects who had follow-up data. The risk of relapse was estimated as the proportion of subjects who reported to be active smokers at the last examination among subjects who reported to be ex-smokers at baseline. The risk of restarting was significantly higher (incidence rate ratio: 2.95, p<0.001) in those who had quit since less than 2 years before their first examination (28.4%) compared to those who had quit since 2 years or more before (9.6%).
