# Supplementary material for: Time and age trends in smoking cessation in Europe
Source: PLoS One. 2019 Feb 7;14(2):e0211976. doi: 10.1371/journal.pone.0211976 (PMC6366773; doi:10.1371/journal.pone.0211976)
Supplement: S3 Table — (DOCX) [file pone.0211976.s008.docx]

**S3 Table. Comparison of the age at smoking initiation and cessation reported at different occasions in multi-wave studies***

|  | 1^st^ wave  (baseline) | 2^nd^ wave  (1^st^ follow-up) | 3^rd^ wave  (2^nd^ follow-up) |
| --- | --- | --- | --- |
| ECRHS clinical (n=393) |  |  |  |
| Year of interview,  median | 1991 | 2001 | 2012 |
| Age at interview |  |  |  |
| Years, mean (SD) | 36.0 (6.2) | 45.0 (6.2) | 56.2 (6.2) |
| Difference from 1^st^ wave, mean (SD) | - | 9.0 (0.7) | 20.2 (0.9) |
| Age at smoking initiation |  |  |  |
| Years, mean (SD) | 16.6 (2.7) | 16.4 (2.7) | 16.3 (2.9) |
| Difference from 1^st^ wave, mean (SD) | - | -0.2 (1.8) | -0.3 (2.0) |
| Spearman correlation coefficient (p-value) |  | 0.77 (<.0001) | 0.74 (<.0001) |
| Age at smoking cessation |  |  |  |
| Years, mean (SD) | 30.5 (6.5) | 31.3 (7.3) | 32.3 (8.5) |
| Difference from 1^st^ wave, mean (SD) | - | 0.8 (4.1) | 1.9 (6.1) |
| Spearman correlation coefficient (p-value) |  | 0.84 (<.0001) | 0.73 (<.0001) |
| ISAYA (n=204) |  |  |  |
| Year of interview,  median | 1999 | 2008 | - |
| Age at interview |  |  |  |
| Years, mean (SD) | 41.9 (6.9) | 50.5 (6.9) | - |
| Difference from 1^st^ wave, mean (SD) | - | 8.6 (0.7) | - |
| Age at smoking initiation |  |  |  |
| Years, mean (SD) | 16.9 (3.2) | 16.8 (3.1) | - |
| Difference from 1^st^ wave, mean (SD) | - | -0.1 (1.6) | - |
| Spearman correlation coefficient (p-value) | - | 0.80 (<.0001) | - |
| Age at smoking cessation |  |  |  |
| Years, mean (SD) | 33.0 (8.0) | 33.4 (8.6) | - |
| Difference from 1st wave, mean (SD) | - | 0.4 (4.2) | - |
| Spearman correlation coefficient (p-value) | - | 0.87 (<.0001) | - |

***** Only subjects who participated in all the waves and consistently reported to be ex-smokers were included.

This table reports the average age at initiation and cessation in subjects who consistently reported to be ex-smokers at baseline and at all the follow-up questionnaires, with the objective of evaluating the extent of bias in our study. The average age at initiation was extremely consistent across different waves: even after 20 years from the first questionnaire, the average difference in the reported age at initiation was less than 15 weeks, suggesting that bias had a minimal influence on our analysis. On the other hand, the difference in age at cessation was about 2 years in ECRHS: this non-negligible difference was likely caused by a subgroup of ex-smokers at baseline who experienced smoking relapses between study waves and who consequently reported a later age at cessation at follow-up.
